# Cell Permeability of Isomeric Macrocycles: Predictions and NMR Studies

**DOI:** 10.1021/acsmedchemlett.1c00126

**Published:** 2021-05-18

**Authors:** Fabio Begnini, Vasanthanathan Poongavanam, Yoseph Atilaw, Mate Erdelyi, Stefan Schiesser, Jan Kihlberg

**Affiliations:** † Department of Chemistry - BMC, Uppsala University, Box 576, 75123 Uppsala, Sweden; ‡ Department of Medicinal Chemistry, Research and Early Development, Respiratory and Immunology (R&I), BioPharmaceuticals R&D, AstraZeneca, Pepparedsleden 1, 43183 Mölndal, Sweden

**Keywords:** Cell permeability, conformational sampling, macrocycle, NMR spectroscopy

## Abstract

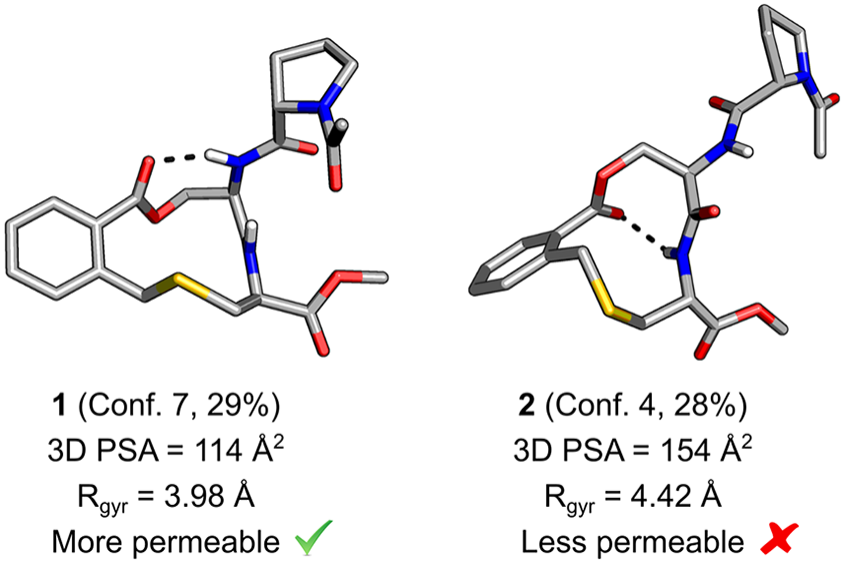

Conformation-dependent 3D descriptors have been shown to provide better predictions of the physicochemical properties of macrocycles than 2D descriptors. However, the computational identification of relevant conformations for macrocycles is nontrivial. Herein, we report that the Caco-2 cell permeability difference between a pair of diastereomeric macrocycles correlated with their solvent accessible 3D polar surface area and radius of gyration. The descriptors were calculated from the macrocycles’ solution-phase conformational ensembles and independently from ensembles obtained by conformational sampling. Calculation of the two descriptors for three other stereo- and regioisomeric macrocycles also allowed the correct ranking of their cell permeability. Methods for conformational sampling may thus allow ranking of passive permeability for moderately flexible macrocycles, thereby contributing to the prioritization of macrocycles for synthesis in lead optimization.

Macrocycles are of major interest in efforts to discover drugs for targets which are challenging to modulate with rule of 5 (Ro5) compliant compounds.^1−3^ In particular, the ability of macrocycles to adopt disk- and sphere-like shapes make them ideal ligands for targets that have large, flat, or groove-shaped binding sites.^2^ It is essential that macrocycles are cell permeable in order to reach intracellular difficult-to-drug targets, such as protein–protein interactions. Cell permeability is also required for compounds to be absorbed after oral administration.^4^ Interestingly, some studies have found that macrocyclization can result in major increases in cell permeability,^5,6^ while others have found only a limited increase^7,8^ or a reduction in permeability upon macrocyclization.^9^

Macrocyclic drugs often require long synthetic routes, and the macrocyclization step may be accompanied by low yields or require significant optimization.^10^ Methods for the prediction of pharmacokinetic properties, such as cell permeability, are therefore of significant interest to reduce the synthesis of macrocycles outside the desired property space. Recent studies have concluded that 2D descriptors for lipophilicity and polarity, for example, cLogP and the topological polar surface area (TPSA), that are often used for design of Ro5 compliant compounds are less useful for macrocycles in particular when their size, structural complexity, and flexibility increases.^11−14^ Instead, knowledge of the 3D structures, conformational preferences, and intramolecular interactions such as hydrogen bonds has been suggested to be essential for better prediction of cell permeability.^13−15^ However, prediction of biologically relevant conformations of complex macrocycles is far from trivial.^14,16^ Some promising progress has been made for small sets of macrocyclic peptides,^5,17,18^ and natural-product-inspired macrocycles,^14,19,20^ albeit by using substantial time and computational resources for each compound. However, in order to impact on the prioritization of compounds for synthesis in lead optimization, medium- to high-throughput methods that are amenable to automation are desired.

Diastereomers share the same 2D descriptors but may differ in cell permeability and other properties that influence oral absorption.^19,21^ Predicting property differences displayed by diastereomers therefore requires use of descriptors derived from their conformations.^14^ Consequently, diastereomers provide ideal opportunities to evaluate methods for generation of structure–property relationships for compounds having complex structures. We recently discovered four diastereomeric macrocycles that display a significant difference in permeability across human colon adenocarcinoma (Caco-2) cell monolayers in a project aimed at finding inhibitors of the Keap1-Nrf2 protein–protein interaction.^22^ Herein, we report the determination of the solution conformational ensembles of the two macrocycles that differed most in permeability. We also report that descriptors calculated from the independent prediction of the conformational ensembles of these two macrocycles ranked their permeability correctly.

The Caco-2 cell line has been used as an *in vitro* model of oral absorption in humans for over 30 years.^23^ Diastereomeric macrocycles **1**–**4**,^22^ which all contain *S*-Pro but differ in the stereochemistry of the Ser and Cys moieties, displayed differences in their passive, efflux inhibited permeabilities. Macrocycle **1** had the highest permeability, which was roughly 7-fold higher than that of **2**, whereas macrocycles **3** and **4** had intermediate permeabilities. As macrocycles **1**–**4** display no or only minor differences in their measured LogD_7.4_ values, NMR studies and conformational sampling were performed for **1** and **2** to investigate the reason for their permeability difference (Figure 1).

**Figure 1:**
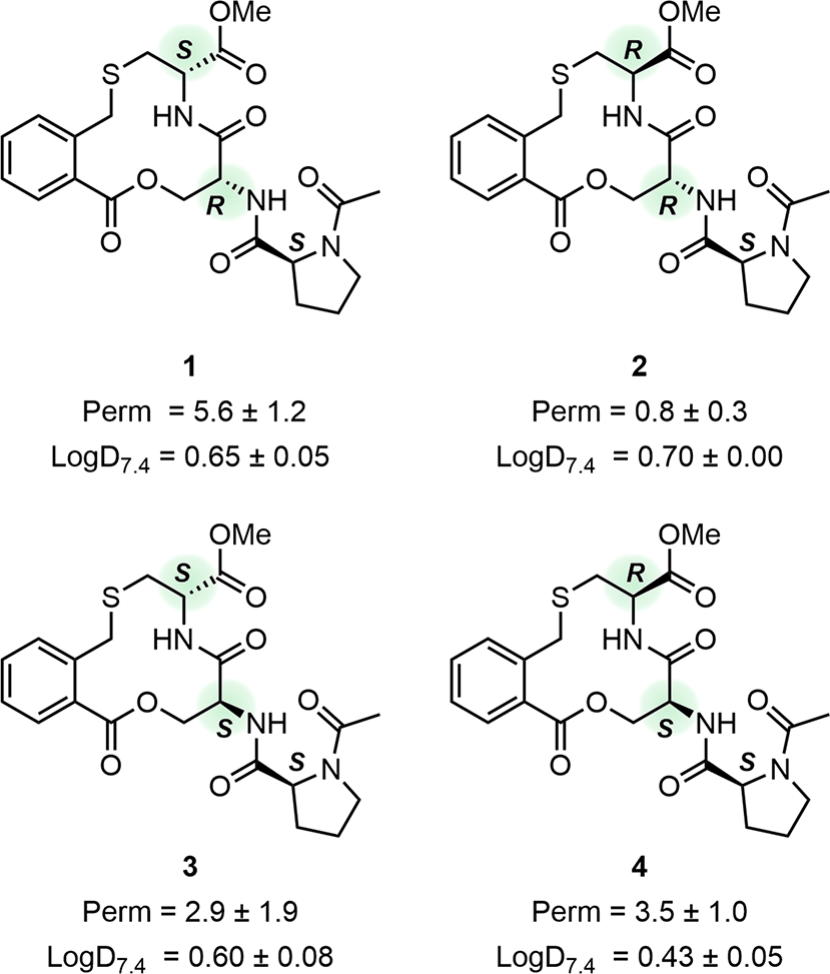
Structures of macrocycles **1**–**4** as well as their passive permeability across a Caco-2 cell monolayer (Perm: P_app_ AB+inhibitor cocktail ×10^–6^ cm/s) and their lipophilicity (LogD_7.4_) determined at pH 7.4. Standard errors were obtained from four and five repeats for **1** (LogD_7.4_ and Caco-2, respectively) and three repeats for each assay for **2**–**4**.

The conformations adopted in a low dielectric medium can be used to predict permeability differences between compounds.^13−15,18,19^ Consequently, we determined the conformational ensembles of macrocycles **1** and **2** in chloroform, which has a similar dielectric constant (ε = 4.8) to that of the interior of a lipid bilayer (ε = 3.0).^24^ As **1** and **2** are expected to exist as ensembles of rapidly interconverting conformations, we used the NMR analysis of molecular flexibility in solution (NAMFIS) algorithm^25^ to deconvolute population averaged NMR data into individual conformations. Previously, this algorithm has been applied to determine the solution ensembles of small molecules, peptides, and macrocycles.^15,16,26,27^

By the use of nuclear Overhauser effects (NOEs), scalar coupling constants, and theoretical conformational ensembles as inputs, NAMFIS generates the ensemble that best fits to the experimental observations (Tables S2–S5, Figure S2). NOEs were quantified from a NOESY buildup with at least four spectra using the initial rate approximation. A pool of theoretically feasible conformers were generated by Monte Carlo conformational searches (Table S6) using five different force fields combined with implicit chloroform and water solvation models for each search, to ensure that the conformational space available for the compounds is sampled efficiently.^15^ For both **1** and **2**, the 10 output ensembles were merged, and redundant conformers were eliminated using a 1 Å root-mean-square deviation (RMSD) cutoff (Table S6). The conformations adopted by the macrocyclic core of **1** and **2** are well-described both by the NMR data and the theoretical conformational search, whereas the orientations of the proline and methyl ester side-chains could not be defined in the NAMFIS analysis because of an insufficient number of experimental data (Tables S2–S5, Figure S2). For the proline moiety of **1** and **2**, the orientations of the Ser C_α_-NH bond were defined by the H_α_-NH coupling constant and by two NOEs between the Ser NH and the hydrogen atoms of the macrocyclic core. However, no or only one experimental restraint was found for the Pro C_α_–CONH and amide bond, just as for the bonds of Cys methyl ester. The lowest energy (OPLS2) conformation about these bonds was therefore predicted by torsional scans conducted with 12 rotamers and 30° angle increments per bond.

The conformational ensemble of **1** consisted of seven conformations (Figure 2A, Table S9). Pairwise RMSD values for all heavy atoms (i.e., all non-hydrogen atoms) in the seven conformations ranged from 0.97 to 2.96 Å (Table S12), while the pairwise RMSD values of heavy atoms of the macrocyclic core ranged from 0.36 to 0.90 Å (Table S10). One conformational family (conformations 1, 3 and 7) and four distinct conformations were identified using a heavy atom RMSD cutoff of 0.5 Å for the heavy atoms in the macrocyclic core. Conformations 1, 3 and 7 also have the proline and methyl ester side chains in similar orientations where the proline moiety is oriented toward the macrocyclic ring. However, the orientation of the carbonyl oxygen of the lactone differ between the three conformations in the family. The macrocycle as well as the side chains in conformation 2 display similarities with conformation 1 but differ from conformations 3 and 7 in the family. In contrast, conformations 4–6 differ significantly from the other four and between each other, both for the macrocyclic core and for the orientation of the two side chains. Conformation 4 does not show any intramolecular hydrogen bond (IMHB), whereas the other six conformations possess either one or two IMHBs (Figure 2A, Table S14).

**Figure 2:**
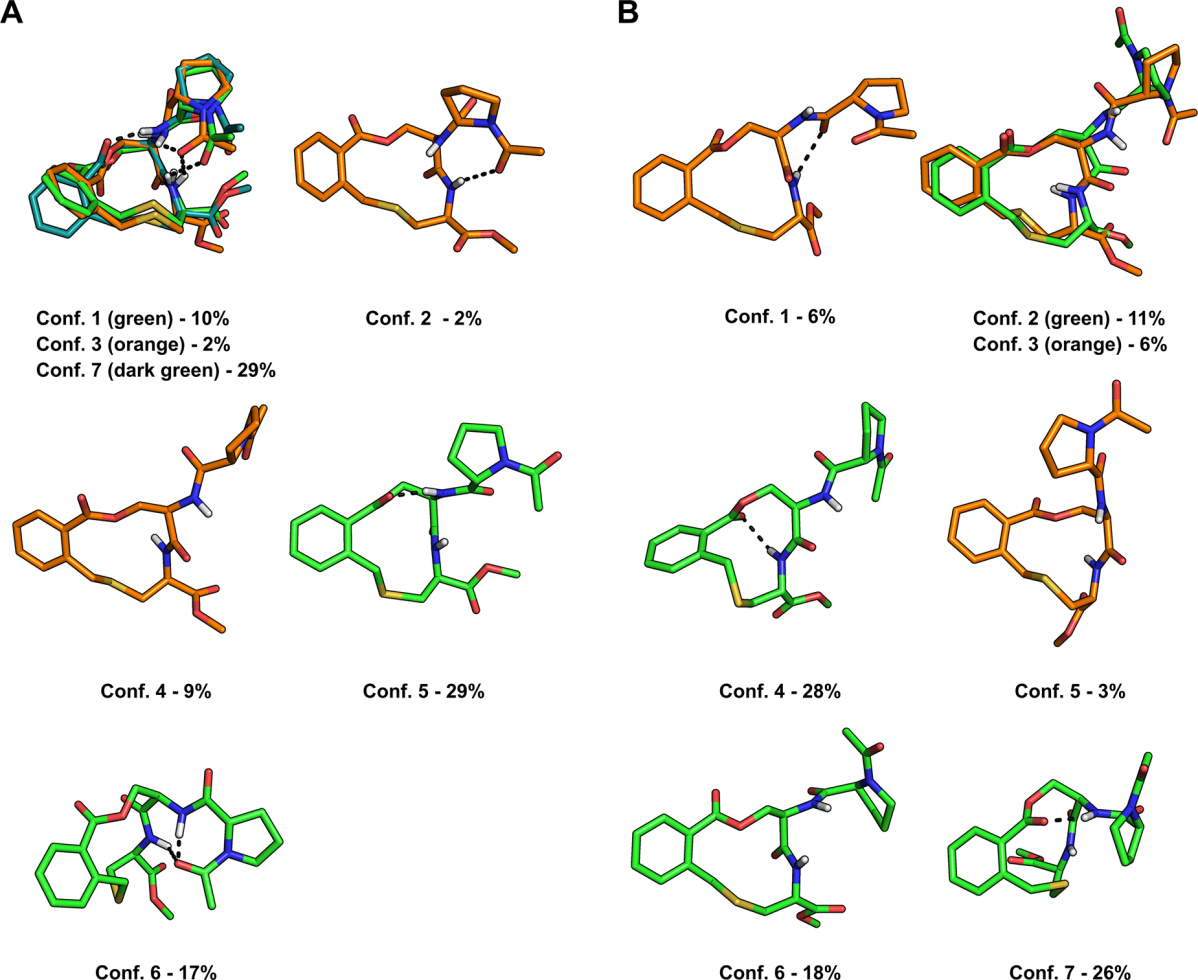
Conformational ensembles of macrocycles **1** (A) and **2** (B) in CDCl_3_ determined by NAMFIS analysis. Major conformations (relative population ≥10%) are colored in green; minor conformations (relative population <10%) are in orange. Conformations that have an RMSD ≤ 0.5 Å for the heavy atoms in the macrocyclic core have been superimposed on the basis of these atoms. IMHBs are indicated as black dashed lines. Nonpolar hydrogen atoms have been omitted for clarity.

Macrocycle **2** also populates seven conformations that show a similar structural diversity as the members of the ensemble of macrocycle **1** (Figure 2B, Table S9). Pairwise RMSD values for all heavy atoms of **2** range from 1.55 to 3.02 Å (Table S13), while the RMSD values for macrocycle core range from 0.33 to 0.99 Å (Table S11). Conformations 2 and 3 cluster into one family when using a heavy atom RMSD cutoff of ≤0.5 Å for the macrocyclic core. These two conformations share the shape of the macrocycle but differ somewhat in the orientation of the amide bond within the macrocyclic core and in the orientation of the two side-chains. The macrocycle ring in conformation 1 has similarities to the ring in conformations 2 and 3, whereas the macrocycles in the remaining conformations (4–7) are distinct from each other and from 1–3 (Figure 2B, Table S11). Single IMHBs are formed in three (1, 4, and 7) of the conformations of **2** (Figure 2B, Table S14).

We performed conformational sampling of **1** and **2** to investigate how well the sampled ensembles resembled the experimental ones obtained by NAMFIS analysis and if the sampled ensembles could be used to predict the permeability difference between **1** and **2**. Conformational sampling was performed using OMEGA^28^ starting from the SMILES (simplified molecular-input line-entry system) codes of **1** and **2** in implicit chloroform (ε = 4.8). An energy window of 10 kcal/mol was used to evaluate to what extent OMEGA identified conformations similar to the experimentally determined ones. Conformations obtained from sampling were energy minimized using the molecular mechanics force field MMFF94.^29^ The number of conformations identified by sampling differed significantly between macrocycles **1** and **2**; eight conformations were found for **1** and 28 for **2**.

Sampling of biologically relevant conformations of macrocycles is far from trivial.^16,30^ Therefore, comparisons of the experimentally determined and sampled ensembles of **1** and **2** were made for all heavy atoms (Figure 3A,B) and for all heavy atoms in the macrocyclic core (Figure 3C,D). Using an RMSD of ≤1.0 Å as the cutoff for high similarity for the whole macrocycle revealed that none of the sampled conformations of **1** and **2** reproduced the experimental conformations with high similarity (Figure 3A,B). However, for **1** the sampled conformations reproduced five of the seven experimental conformations (numbers 1, 3, 5–7; 89% of the ensemble) with intermediate similarity (1.0–1.5 Å), with the sampled minimum energy conformation (MEC) being similar to three of the experimental conformations. For **2**, the sampled conformations reproduced four of the seven experimental conformations (numbers 1, 3, 4 and 6; 58% of the ensemble) with intermediate similarity, but at energies >5 kcal/mol above the MEC. A principle moment of inertia (PMI) plot revealed that experimental and sampled conformations of **1** and **2** adopted similar rod to sphere-like shapes (Figure S4). Using an RMSD ≤ 0.5 Å as cutoff for high similarity revealed that the structure of the macrocyclic core in all but conformation 4 (9%) of **1**, and conformations 1 (6%) and 2 (11%) of **2**, were reproduced by at least one of the sampled conformations (Figure 3C,D). Interestingly, three of the core conformations in each of the ensembles of **1** and **2**, which represent 75 and 37% of each ensemble, where reproduced with excellent similarity (RMSD ≤ 0.1 Å). However, most experimental conformations were reproduced by sampled conformations having energies close to or >5 kcal/mol above the MECs. Only the core of conformation 7 of macrocycle **1** was reproduced with excellent similarity by the sampled MEC. Thus, sampling was less successful in generating ensembles in which the side-chains adopted correct conformations than in identifying the solution conformations of the macrocyclic cores. In addition, sampled conformations that were similar to the experimentally determined ones were usually found at energies ≥5 kcal/mol above the MEC. This, again, illustrates the difficulty of force fields, such as MMFF94, to rank the conformations for macrocycles by energy so that the major solution conformations are identified.^14,16^

**Figure 3:**
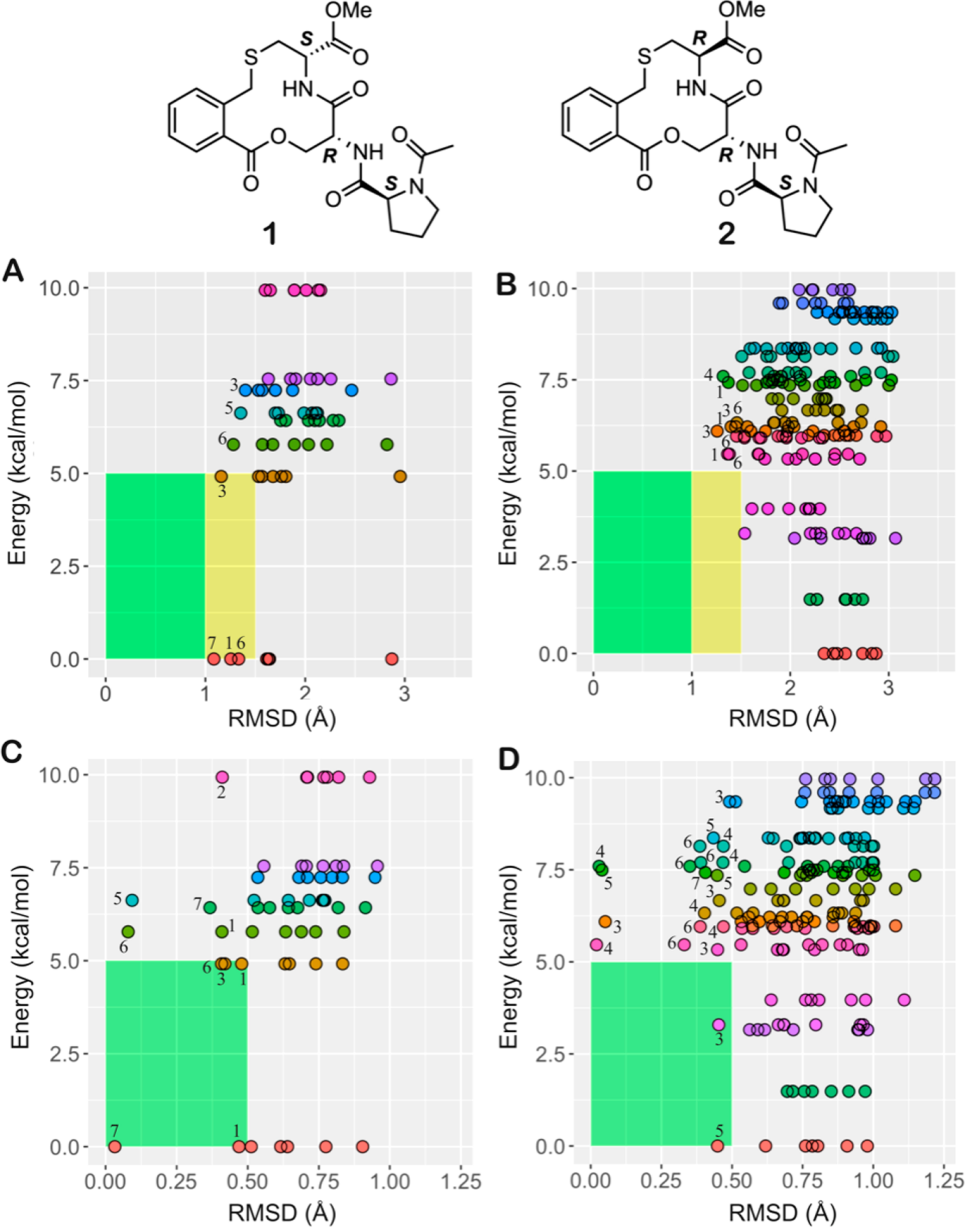
Comparison of all heavy atoms (A and B), or the heavy atoms of the macrocyclic core (C and D), in the sampled conformations of **1** and **2** to the experimentally determined conformations. In each panel, the sampled conformations are arranged by increasing energy above the minimum energy conformation (MEC), as indicated by the horizontal rows of colored circles. The circles indicate how similar (by RMSD) the sampled conformation at that energy is to each of the seven experimentally determined conformations of **1** or **2**. In panels A and B, the numbers of all experimental conformations that are similar (RMSD ≤ 1.5 Å, for all heavy atoms in **1** and **2**) to the sampled conformations are given adjacent to the corresponding colored circle. In panels C and D, the numbers of all experimental conformations in which the macrocyclic core is similar (RMSD ≤ 0.5 Å, for all heavy atoms in the macrocyclic ring of **1** and **2**) to the ring in the sampled conformations are given adjacent to the corresponding colored circle. In panels A–D, the space in which the experimental and sampled conformations have a high similarity (RMSD ≤ 1.0 Å for all heavy atoms, RMSD < 0.5 Å for the heavy atoms in the macrocyclic core) within 5 kcal/mol of the MEC is highlighted in green. Similarly, in panels A and B, the space in which the experimental and sampled conformations have medium similarity (RMSD 1.0–1.5 Å for all heavy atoms) within 5 kcal/mol of the MEC is highlighted in yellow.

Recently, we suggested that physicochemical properties of macrocycles are better assessed by molecular descriptors, such as the radius of gyration (*R*_gyr_)^31^ and solvent accessible 3D polar surface area (SA 3D PSA)^13,32^ than by energy- or RMSD-based criteria.^16^ We therefore calculated the *R*_gyr_ and SA 3D PSA for both the experimentally determined and sampled ensembles of **1** and **2** to investigate if these descriptors correlate with the observed permeability difference between the compounds. *R*_gyr_ is suitable to describe differences in size between diastereomers as it depends on the 3D structure and conformation(s) of a compound. The topological polar surface area (TPSA) is a satisfactory descriptor of polarity for Ro5 compliant compounds^33^ but does not account for differences between diastereomers. For these, it is better to use the solvent-accessible 3D PSA^32^ (SA 3D PSA) and to include partially charged atoms in addition to nitrogen, oxygen, and attached hydrogen atoms^34,35^ in the calculations.^13^

The *R*_gyr_ was calculated for the experimentally determined conformations of macrocycles **1** and **2**, as well as for the sampled ensembles (Figure 4, Tables S14–S15). The population weighted mean *R*_gyr_ is somewhat smaller for the experimental conformations of macrocycle **1** than for **2** (4.04 vs 4.23 Å). Even though this difference may not have a major impact on the observed difference in permeability, it is interesting to note that the mean *R*_gyr_ for the sampled conformational ensembles of **1** and **2** (4.01 vs 4.27 Å) agreed well with the values obtained for the experimentally determined ensembles. Calculation of the SA 3D PSA revealed that the population weighted mean SA 3D PSA was significantly lower for the experimentally determined ensemble of **1** than for the ensemble of **2** (132 vs 146 Å, Figure 4B). This agrees well with the ensemble of **1** showing a higher degree of intramolecular hydrogen bonding than that of **2** (Figure 2, Table S14). The SA 3D PSA for the sampled conformational ensembles of **1** and **2** (means; 134 vs 144 Å) showed an excellent agreement with the values from the experimental ensembles, just as was found for R_gyr_. In conclusion, the experimental ensemble of **1** is shifted toward lower values for both R_gyr_ and SA 3D PSA than the ensemble of **2**, in full agreement with **1** displaying a higher passive Caco-2 cell permeability than **2**. Importantly, these differences between **1** and **2** were also well predicted by the ensembles sampled by OMEGA, suggesting that calculated descriptors from conformational sampling could be used for prospective ranking of cell permeability.

**Figure 4:**
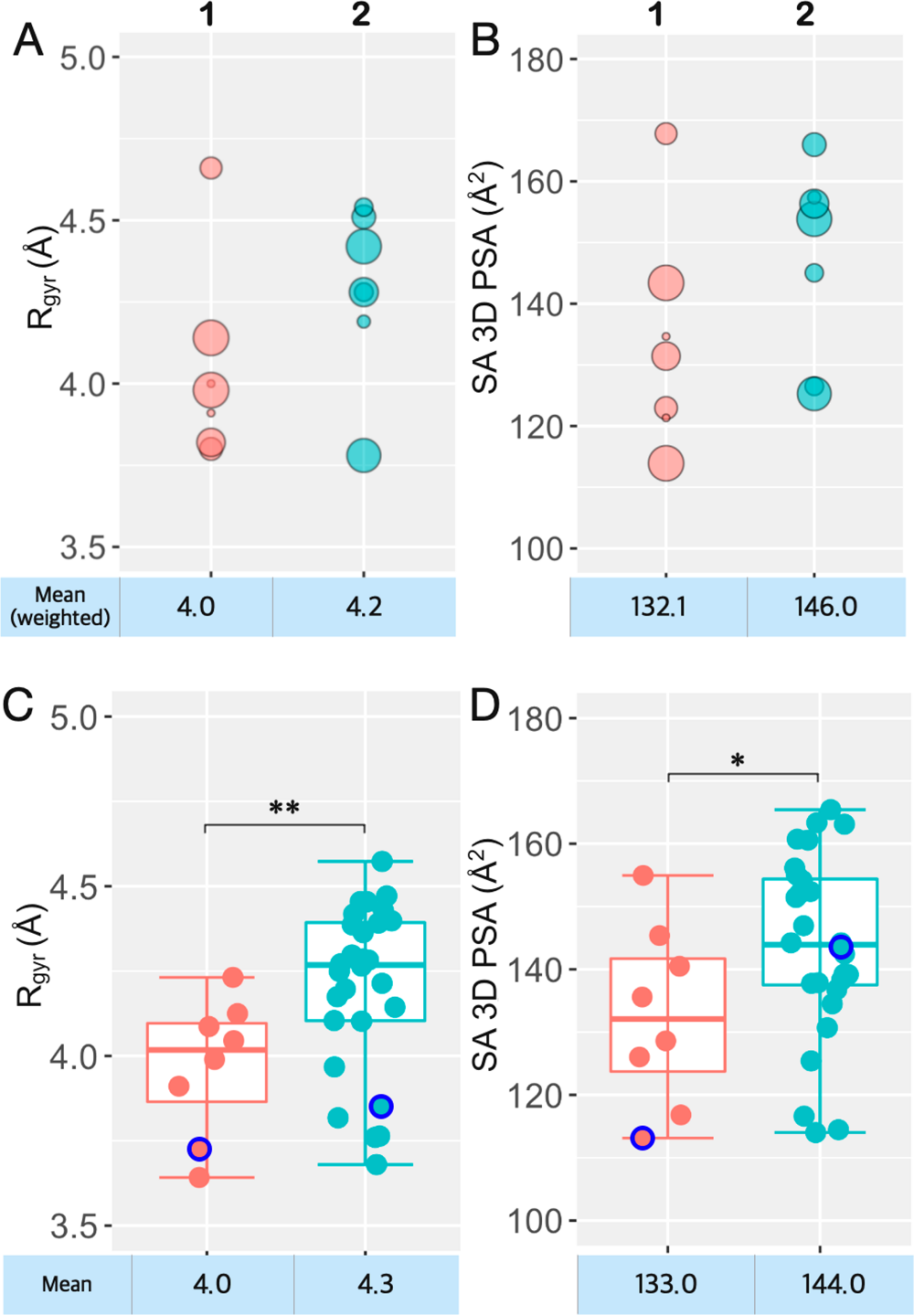
(A) Calculated radius of gyration (*R*_gyr_) and (B) solvent-accessible 3D polar surface area (SA 3D PSA) for the experimentally determined conformations of macrocycles **1** and **2**. The size of each circle is proportional to the relative population of each conformer. (C) Calculated radius of gyration (*R*_gyr_) and (D) solvent-accessible 3D polar surface area (SA 3D PSA) for the sampled conformational ensembles of macrocycles **1** and **2**. Boxplots show the 50th percentiles as horizontal bars, the 25th and 75th percentiles as boxes, the 25th percentile minus 1.5× the interquartile range and the 75th percentile plus 1.5× the interquartile range as whiskers. The minimum energy conformations (MECs) are indicated as blue circles. Population weighted mean values are given below panels A and B, while mean values are given below panels C and D. Wilcoxon test *p*-values: * ≤ 0.05, ** ≤ 0.01.

In addition, we investigated if predicted 3D descriptors may be used for prospective ranking of cell permeability using macrocycles **5**–**7**,^19^ which include a regioisomeric (**5** and **7**) and a diastereomeric (**6** and **7**) matched pair that differ in passive cell permeability (Figure 5A) but have identical calculated 2D descriptors (Table S17). Sampled ensembles were generated for **5**–**7**, and then *R*_gyr_ and SA 3D PSA were calculated for the conformers, using the same protocols as for **1** and **2**. Encouragingly, the calculated values of the two 3D descriptors agreed well with the permeabilities of **5**–**7**, that is, the least permeable regioisomer **5** had a significantly higher *R*_gyr_ and SA 3D PSA than stereoisomers **6** and **7** (Figure 5B,C). There was also a difference in SA 3D PSA between **6** and **7** that matched the lower permeability of **7**. The less permeable regioisomer **5** adopted more rod-like conformations than the sphere-like **6** and **7** (Figure S4).

**Figure 5:**
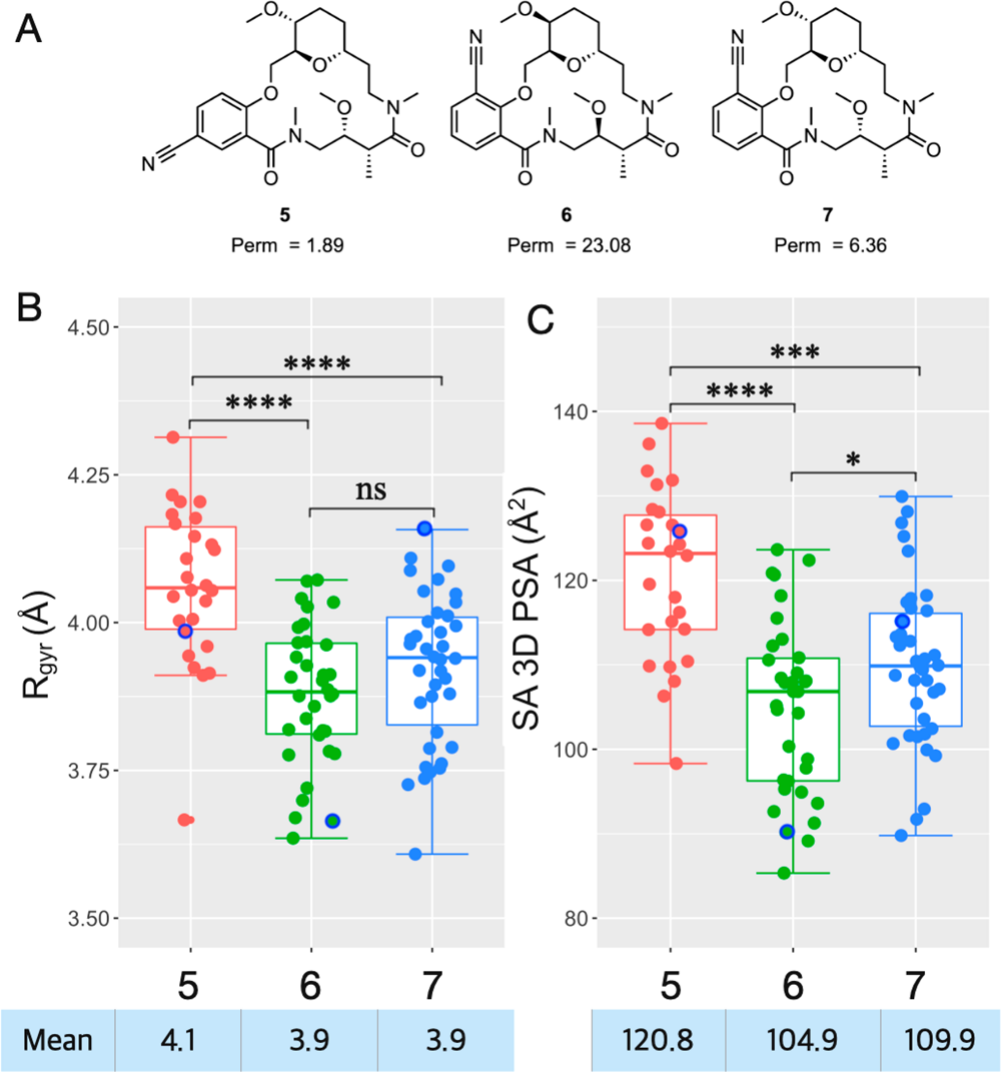
(A) Structures of macrocycles **5**–**7** and their passive permeability across a Caco-2 cell monolayer^19^ (Perm: P_app_ AB+inhibitor cocktail ×10^–6^ cm/s). (B) Calculated radius of gyration (*R*_gyr_) and (C) solvent-accessible 3D polar surface area (SA 3D PSA) for the sampled conformational ensembles of **5**–**7**. Boxplots show the 50th percentiles as horizontal bars, the 25th and 75th percentiles as boxes, the 25th percentile minus 1.5 × the interquartile range and the 75th percentile plus 1.5 × the interquartile range as whiskers. The minimum energy conformations (MECs) are indicated as blue circles, and mean values are given below the panels. Wilcoxon test *p*-values: * ≤ 0.05, ** ≤ 0.01, *** ≤ 0.001, **** ≤ 0.0001.

The flexibility of a compound is another key determinant of its oral bioavailability and cell permeability.^36,37^ Kier’s flexibility index (Φ)^38^ has been highlighted to provide a better description of the flexibility of macrocyclic compounds^36^ than the number of rotatable bonds (NRotB),^37^ which is calculated from single bonds that are not part of a ring. Potentially, use of Φ to characterize the flexibility of the macrocyclic ring, in combination with NRotB for the flexibility of the side chains, could be the preferred approach for macrocyclic compounds. Calculations using the MOE software revealed that **1** and **2** had NRotB = 6 and Φ = 7.12, while **5**–**7** had NRotB = 3 and Φ = 9.32. A value of 10 for Φ was recently proposed as an upper limit within which it could be possible to predict macrocycle cell permeability based on conformational sampling.^14^ It is therefore interesting to note that descriptors calculated for conformational ensembles of **1** and **2**, and **5**–**7**, successfully ranked their permeabilities.

The 3D structure and conformational preferences of compounds that have high structural complexity and/or flexibility are key determinants of their cell permeability.^13,19,36^ Prediction of properties from relevant conformational ensembles would therefore be useful in lead optimization of such compounds. Herein, we found that descriptors, that is, the solvent accessible 3D polar surface area (SA 3D PSA) and radius of gyration (*R*_gyr_), calculated from the conformational ensembles determined by NMR spectroscopy in chloroform correlated with the difference in Caco-2 cell permeability between diastereomeric macrocycles **1** and **2**. This reiterates the relevance of conformation-dependent descriptors for prediction of macrocycle cell permeability.^13,32^ Conformational sampling reproduced the conformations of the macrocyclic cores in the experimental ensembles of **1** and **2** with high or excellent accuracy (RMSD 0.1–0.5 Å), but the conformations of the overall compounds were only reproduced with intermediate accuracy (RMSD 1.0–1.5 Å). However, as previously observed,^16^ the energies of the relevant sampled conformations were usually high above the minimum energy conformation, preventing an energy-based identification of the permeating conformations. Interestingly, the mean values of the SA 3D PSA and *R*_gyr_ for the sampled ensembles of **1** and **2** were in excellent agreement with the corresponding values for the experimental ensembles. This indicates that 3D descriptors from sampled ensembles may be of use for prospective ranking of cell permeability; a hypothesis that was supported by the predicted SA 3D PSA and *R*_gyr_ of the stereo- and regioisomeric macrocycles **5**–**7**. In a recent analysis, we postulated that conformational sampling was likely to provide successful rankings of the permeability for compounds possessing a Kier flexibility index (Φ) < 10.^14^ Our current findings agree well with these results as macrocycles **1** and **2** have Φ = 7.12 and **5**–**7** have Φ = 9.32. Medium-throughput methods for conformational sampling may thus allow ranking of passive permeability for moderately flexible macrocycles. Incorporation of such methods in compound design during the lead optimization phase should help medicinal chemists to select and synthesize only the macrocycles which are within the desired property space.

## Supporting Information Available

NMR data; Monte Carlo molecular mechanics conformational search and NAMFIS conformational analysis of macrocycles **1** and **2**; computational characterization of **1**, **2**, and **5**–**7**; procedures for determination of LogD_7.4_ and Caco-2 cell permeability; purity analyses for **1**–**4**; NMR spectra of **1** and **2** (PDF)

## ABBREVIATIONS

IMHB: intramolecular hydrogen bond
NAMFIS: NMR analysis of molecular flexibility in solution
NRotB: number of rotatable bonds
RMSD: root-mean-square deviation
R_gyr_: radius of gyration
SMILES: simplified molecular-input line-entry system
SA 3D PSA: solvent accessible 3D polar surface area
TPSA: topological polar surface area
