# Supplementary material for: Cell Permeability of Isomeric Macrocycles: Predictions and NMR Studies
Source: ACS Med Chem Lett. 2021 May 18;12(6):983–90. doi: 10.1021/acsmedchemlett.1c00126 (PMC8201747; doi:10.1021/acsmedchemlett.1c00126)
Supplement: Supplementary file 1 — ml1c00126_si_001.pdf [file ml1c00126_si_001.pdf]

Cell Permeability of Isomeric Macrocycles: Predictions and NMR studies

Fabio Begnini,[a,#] Vasanthanathan Poongavanam,[a,#] Yoseph Atilaw,[a] Mate Erdelyi,[a] Stefan Schiesser[b] and Jan Kihlberg[a,*]

[a]Department of Chemistry - BMC, Uppsala University, Box 576, 75123 Uppsala, Sweden

[b]Department of Medicinal Chemistry, Research and Early Development, Respiratory & Immunology (R&I), BioPharmaceuticals R&D, AstraZeneca, Pepparedsleden 1, 43183 Mölndal, Sweden

*Corresponding author

jan.kihlberg@kemi.uu.se, ORCID 0000-0002-4205-6040

[#]Equally contributing authors

Contents

^{1}H NMR signal assignment of compound **1** and **2** 3

NOE Buildups, distances and *J* couplings 4

Monte Carlo molecular mechanics (MCMM) conformational search 9

NAMFIS analysis 10

Sidechain refinement 15

Conformational sampling 15

Calculation of 3D-dependent properties 16

Principal Moments of Inertia plots 20

Cell permeability measurement 21

$LogD_{7.4}$ determination 21

Note on synthesis, characterization and purity 21

NMR spectra 24

^{1}H NMR signal assignment of compound 1 and 2

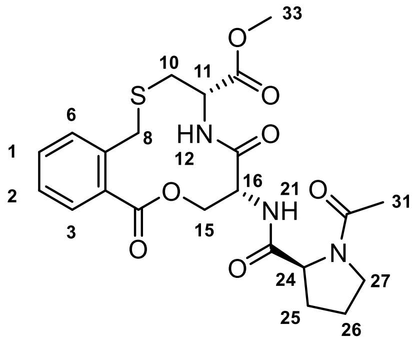

Compound **1**

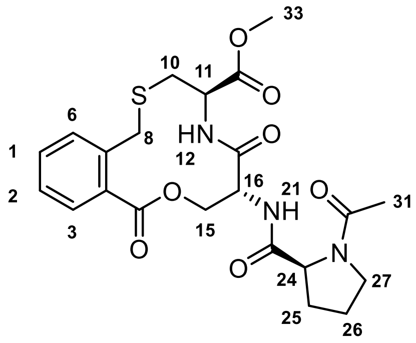

Compound **2**

Figure S1. Structure of compounds **1** and **2** with the corresponding enumeration used for assignment of the ^{1}H NMR signals. The assignment of the protons (Table S1) for compounds **1** and **2** (Figure S1) in $CDCl_3$ was performed using 1D (^{1}H) and 2D (TOCSY and NOESY) NMR spectra recorded at 25 °C on a 800 MHz BRUKER Avance III HD NMR spectrometer equipped with a TCI cryogenic probe.

Table S1. ^{1}H NMR assignment (δ in ppm) of compounds **1** and **2** in $CDCl_3$

No.	δ of **1**	δ of **2**	No.	δ of 1	δ of **2**
3	7.94	7.82	8''	4.33	4.13
12	7.80	7.27	8'	3.97	4.11
21	7.74	7.61	33-Me	3.75	3.77
1	7.49	7.46	27''	3.74	3.64
6	7.43	7.33	27'	3.52	3.48
2	7.36	7.35	10''	3.25	3.23
15''	5.13	4.73	10'	2.98	3.17
11	4.95	4.69	25'', 26''	2.35 - 2.20	2.33, 2.16
16	4.91	4.88	31-Me	2.12	2.13
15'	4.49	4.51	25', 26'	2.03 - 1.93	2.06 - 1.95
24	4.46	4.54			

NOE Buildups, distances and *J* couplings

NOESY spectra were recorded without solvent suppression with alternated mixing times of 700, 600, 500, 400, 300, 200 and 100 ms. The relaxation delay was set to 2.5 s, 16 scans were recorded with 2048 points in the direct dimension (F2) and 512 points in the indirect dimension (F1). NOE intensities were calculated by normalization of the integrals of both cross peaks and diagonal peaks of protons *a* and *b*, according to Equation 1.

$$\mathrm{NOE} = \left[\frac{(\text{cross peak } a * \text{cross peak } b)}{(\text{diagonal peak } a * \text{diagonal peak } b)}\right]^{1/2}$$

Equation 1

Seven normalized NOE intensities were obtained from the different mixing times. Only normalized NOE intensities with at least four consecutive mixing times giving a linear initial NOE rate ($R^2 \geq 0.95$) were used to determine buildup rates (σ), according to Equation 2.

$$r_{ab} = r_{ref}\left(\frac{\sigma_{ref}}{\sigma_{ab}}\right)^{1/6}$$

Equation 2

The distance between protons *a* and *b* (in Ångström) is indicated by r_{ab} while r_{ref} is the reference distance. The distance between geminal methylene protons (1.78 Å) was used as reference. The buildup rate of the reference protons is indicated by σ_{ref} while σ_{ab} is the buildup rate of protons *a* and *b*. Interproton distances and experimentally determined J couplings are reported in Tables S2-5 and summarized in Figure S2.

Table S2. Interproton distances for compound **1**, derived from NOE build-up measurements in $CDCl_3$; (δ in ppm).

Dis. No.	Atom Type	Proton *a*	Proton *b*	δ(^{1}H)*a*	δ(^{1}H)*b*	σ	R^2	Dis. r_{ab} [Å]
1	NHNH	12	21	7.80	7.74	5.3553E-05	0.98	2.49
2	NHCH	12	11	7.80	4.95	2.0238E-05	0.99	2.93
3	NHCH	12	8''	7.80	4.33	2.1267E-05	0.99	2.90
4	NHCH	12	10'	7.80	2.98	1.1776E-05	0.98	3.20
5	NHCH	21	16	7.74	4.91	1.2026E-05	0.99	3.19
6	NHCH	21	15'	7.74	4.49	5.4590E-05	0.99	2.48
7	NHCH	21	8''	7.74	4.33	1.6590E-05	0.99	3.03
8	CHCH	6	8''	7.43	4.33	2.8211E-05	0.99	2.77
9	CHCH	6	8'	7.43	3.97	2.3450E-05	0.99	2.86
10	CHCH	15''	16	5.13	4.91	3.6303E-05	0.99	2.65
11	CHCH	11	8'	4.95	3.97	2.0588E-05	0.99	2.92
12	CHCH	16	15'	4.91	4.49	2.9756E-05	0.98	2.74
13	CHCH	8''	10''	4.33	3.25	1.8826E-05	0.99	2.96
14	CHCH	8'	10''	3.97	3.25	8.4757E-06	0.95	3.38
15	CHCH	8'	10'	3.97	2.98	9.9692E-06	0.99	3.29
Ref	CHCH	15''	15'	5.13	4.49	3.9967E-04	0.98	1.78

Table S3. Interproton distances for compound **2**, derived from NOE build-up measurements in $CDCl_3$; (δ in ppm).

Dis. No.	Atom Type	Proton *a*	Proton *b*	$\delta(^1H)a$	$\delta(^1H)b$	σ	R^2	Dis. r_{ab} [Å]
1	NHCH	21	24	7.61	4.54	4.7549E-05	0.99	2.47
2	NHCH	21	15'	7.61	4.51	3.1140E-05	0.99	2.65
3	NHCH	21	16	7.61	4.88	1.1052E-05	0.99	3.15
4	NHCH	12	11	7.27	4.69	2.3529E-05	0.99	2.78
5	NHCH	12	16	7.27	4.69	2.8539E-05	0.99	2.69
6	NHCH	12	15"	7.27	4.88	1.0221E-05	0.99	3.19
7	CHCH	16	15'	4.88	4.51	1.7588E-05	0.95	2.91
Ref.	CHCH	15"	15'	4.73	4.51	3.3841E-05	0.97	1.78

Table S4. $^3J_{ab}$ vicinal coupling constants for compound **1**in $CDCl_3$

^{3}J No.	Proton *a*	Proton *b*	$\delta(^1H)a$	$\delta(^1H)b$	$^3J_{ab}$ [Hz]
1	11	10'	4.95	2.98	4.5
2	11	10"	4.95	3.25	9.0
3[a]	12	11	7.80	4.95	8.8
4[a]	21	16	7.74	4.91	8.9
5	16	15'	4.91	5.13	2.0
6	16	15"	4.91	4.49	2.9

[a] $^3J_{N\underline{H}-C\underline{H}}$

Table S5. $^3J_{ab}$ vicinal coupling constants for compound **2** in CDCl3

^{3}J No.	Proton *a*	Proton *b*	$\delta(^1H)a$	$\delta(^1H)b$	$^3J_{ab}$ [Hz]
1[a]	21	16	7.61	4.88	7.4
2	16	15"	4.88	4.73	4.4
3	16	15'	4.88	4.51	7.3
4	11	10"	4.69	3.23	4.8
5	11	10'	4.69	3.17	6.7

[a] $^3J_{N\underline{H}-C\underline{H}}$

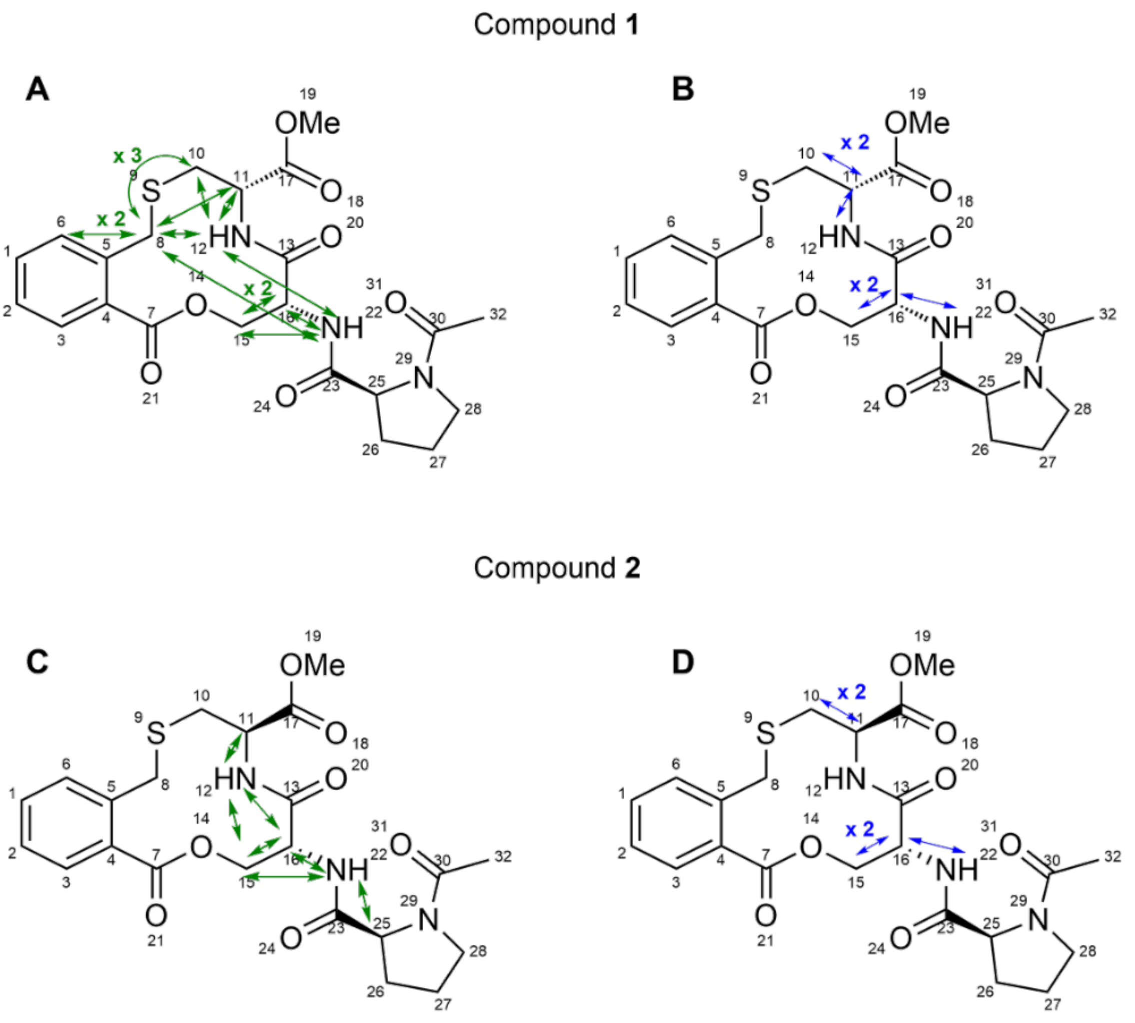

Figure S2. Summary of experimentally determined distances and *J* couplings. (**A**) Experimentally determined distances for compound **1**. (**B**) Experimentally determined *J* couplings for compound **1**. (**C**) Experimentally determined distances for compound **2**. (**D**) Experimentally determined *J* couplings for compound **2**.

Monte Carlo molecular mechanics (MCMM) conformational search

Theoretical conformational ensembles for compounds **1** and **2** were obtained by performing a MCMM conformational search using the software Macromodel BatchMin V12.1 as implemented in the Schrödinger package. The conformational search was done using five different force fields (AMBER*, MMF, OPLS, OPLS-2005 and OPLS3e), each with the Generalized Born/Surface Area (GB/SA) solvation models for chloroform and water. The Monte Carlo algorithm was used with intermediate torsion sampling, 50 000 steps and an RMSD cut-off = 2Å. The conformations were energy minimized using Polak-Ribière Conjugate Gradient (PRCG) with a maximum of 5000 iterative steps and those within 42 kJ/mol from the global minimum were saved (Table S6). All the obtained conformations were combined and subjected to redundant conformer eliminations (RCE) by comparison of heavy atoms coordinates with an RMSD cut-off = 1Å, providing the final ensemble employed in the NAMFIS analysis.

Table S6. Result of the MCMM conformational search for compound **1** and **2**.

Solvation model	Force field	Compound **1**	Compound **2**
	AMBER*	13	15
	MMFF	10	18
**CHCl3**	OPLS	12	14
	OPLS3E	5	14
	OPLS-2005	11	17
	AMBER*	12	13
	MMFF	11	14
**H_2O**	OPLS	8	10
	OPLS3E	10	14
	OPLS-2005	11	14
	Total[a]	103	143
	RCE[b]	46	73

[a]Total unique conformations found.

[b]Conformations obtained after redundant conformation elimination (RCE) with the root-mean-square deviation cutoff set to 1Å for heavy atoms.

NAMFIS analysis

Molar fractions of conformations of compounds **1** and **2** in $CDCl_3$ were determined using the NMR analysis of molecular flexibility in solution (NAMFIS) algorithm. NAMFIS is a method that uses experimentally assigned distances and coupling constants and fits them to back-calculated values of computationally generated conformations.[1, 2] To determine interproton distances and ^3J couplings for the theoretical ensembles, the MCMM generated computational conformations were analyzed and the respective distances and dihedrals angles were measured. Vicinal ^3J coupling constants were calculated using the generalized form of the Karplus equation,[3, 4] shown in Equation 3: φ is the dihedral angle between two ^1H nuclei separated by three bonds, A = 9.4, B = -1.1 and C = 0.4.

$$^3J_{HH}(\varphi) = \mathrm{A}\cos^2(\varphi) + \mathrm{B}\cos(\varphi) + \mathrm{C}$$

Equation 3

The results of the NAMFIS analyses were validated by evaluating the variation of the conformational restraints upon addition of 10% random noise to the experimental distances, by the random removal of 10% of individual restrains and by comparison of the experimentally observed and back-calculated distances. The results of the NAMFIS analysis are given in Tables S7-13.

Table S7. Experimentally determined and back-calculated (NAMFIS) interproton distances (Å) and *J* couplings for compound **1**.

Dis. No.	Exp.	Calc.	^{3}J No.	Exp.	Calc.
1	2.49	2.33	1	4.5	3.7
2	2.93	2.81	2	9.0	8.3
3	2.90	2.81	3	8.8	8.9
4	3.20	2.91	4	8.9	7.5
5	3.19	2.92	5	2.0	2.6
6	2.48	2.73	6	2.9	3.1
7	3.03	2.98			
8	2.77	2.68			
9	2.86	2.79			
10	2.65	2.45			
11	2.92	2.87			
12	2.74	2.55			
13	2.96	2.95			
14	3.38	3.35			
15	3.29	3.19			
RMSD	0.16		RMSD	0.78	

Table S8. Experimentally determined and back-calculated (NAMFIS) interproton distances (Å) and *J* couplings for compound **2**.

Dis. No.	Exp.	Calc.	^{3}J No.	Exp.	Calc.
1	2.47	2.55	1	7.4	7.2
2	2.65	2.63	2	4.4	3.8
3	3.15	2.91	3	7.3	7.1
4	2.78	2.61	4	4.8	4.5
5	2.69	2.55	5	6.7	6.7
6	3.19	3.21			
7	2.91	2.81			
RMSD	0.13		RMSD	0.34	

Table S9. Solution ensemble determined by NAMFIS for compound **1** and **2**.

Compound **1**		Compound **2**	
Conf. No.	% [a]	Conf. No.	% [a]
1	10	1	6
2	2	2	11
3	2	3	6
4	9	4	28
5	17	5	3
6	29	6	18
7	29	7	26

[a] Percentage population of the indicated conformer in solution, as determined by NAMFIS analysis. Conformers having populations ≤1% have been discarded.

Table S10. Heavy atom RMSD (macrocyclic core only) for compound **1**.

Conf. No.	1	2	3	4	5	6
1	0	0	0	0	0	0
2	0.36	0	0	0	0	0
3	0.37	0.59	0	0	0	0
4	0.70	0.75	0.82	0	0	0
5	0.74	0.80	0.68	0.83	0	0
6	0.73	0.80	0.74	0.55	0.75	0
7	0.46	0.61	0.51	0.77	0.90	0.64

Table S11. Heavy atom RMSD (macrocyclic core only) for compound **2**.

Conf. No.	1	2	3	4	5	6
1	0	0	0	0	0	0
2	0.54	0	0	0	0	0
3	0.59	0.33	0	0	0	0
4	0.70	0.91	0.99	0	0	0
5	0.76	0.83	0.88	0.86	0	0
6	0.78	0.77	0.80	0.98	0.76	0
7	0.95	0.88	0.98	0.72	0.79	0.99

Table S12. Heavy atoms RMSD (all) for compound **1**.

Conf- No.	1	36	42	18	41	40
1	0	0	0	0	0	0
2	1.25	0	0	0	0	0
3	1.18	1.45	0	0	0	0
4	2.96	2.46	2.82	0	0	0
5	1.70	1.41	1.89	2.19	0	0
6	1.28	1.62	1.19	2.82	2.04	0
7	0.97	1.27	1.38	2.49	1.16	1.55

Table S13. Heavy atoms RMSD (all) for compound **2**.

Conf. No.	1	2	3	4	5	6
1	0	0	0	0	0	0
2	2.25	0	0	0	0	0
3	1.93	1.76	0	0	0	0
4	1.88	1.74	1.55	0	0	0
5	1.66	2.30	2.75	2.37	0	0
6	3.02	1.65	2.21	2.29	2.66	0
7	2.92	2.14	2.90	2.43	1.64	2.44

Sidechain refinement

The torsional scanning approach was used to predict the orientations of the side-chains of compounds **1** and **2**, as the Pro Cα–CONH, Cys Cα–CO_2Me and C(=O)-OMe bonds, as well as the N-acetyl amide bond are not well defined by the NMR data (Figure S3).

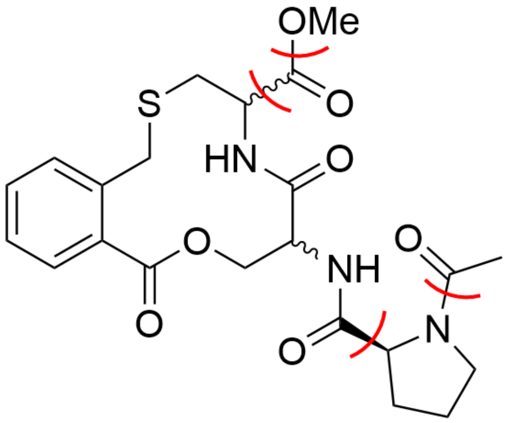

Figure S3. Bonds included in the torsional scanning.

The Rapid Torsion Scan tool from the Schrodinger software[5] was used for each of the seven experimental conformations obtained for **1** and **2** by the NAMFIS approach. 12 rotamers of each of the four bonds (Figure S3) in each experimental conformation was scanned with $\geq$30° angle increments per bond. The conformation with the lowest total energy for each bond, as calculated by OPLS2, was selected and the torsional angles adjusted in corresponding conformation. This provided seven optimized conformations for each of **1** and **2**.

Conformational sampling

Conformational sampling was performed using the distance geometry-based OMEGA tool (Version 3.1.2.2),[7] starting from the SMILES (Simplified Molecular-Input Line-Entry System) codes of macrocycles **1** and **2**. The Sheffield implicit solvation model for chloroform ($\varepsilon = 4.8$)[8] was used to mimic the membrane environment. Further, the following settings were used for the conformational sampling: energy window (10 kcal/mol), elimination of duplicate conformer threshold (RMSD, 0.75 Å), the maximum number of iterations (2 000 steps), and force field (MMFF94s)[9].

Calculation of 3D-dependent properties

The number of intramolecular hydrogen bonds (IMHBs) and the radius of gyration (R_{gyr}) were calculated for each experimental and sampled conformation of **1**, **2** and **5**-**7** using the Schrödinger suite[5] and MOE software[10], respectively (Tables S14-16). Absolute partial charges were calculated for each conformation using the B3LYP/6-31G** basis set in the Jaguar tool,[6] available in the Schrödinger suite. The PB solvation model with ε=4.8 and a probe radius of 2.52 Å for chloroform was used. The solvent-accessible three-dimensional polar surface area (SA 3D PSA) was then calculated with PyMol (Version 2.1) from the solvent-accessible surface area defined using a solvent probe radius of 1.4 (Tables S14 and S15). Other settings for calculating the SA 3D PSA, including the partial charge cut-off, have been described previously.[11, 12]

Table S14. Summary of calculated 3D dependent molecular descriptors of the experimentally determined conformations of compounds **1** and **2**.

**1**					**2**				
Conf. No.	%[a]	IMHBs[b]	R_{gyr}[c] (Å)	SA 3D PSA[d] (Å^2)	Conf. No.	%[a]	IMHBs[b]	R_{gyr}[c] (Å)	SA 3D PSA[d] (Å^2)
1	10	1	3.80	123.0	1	6	1	4.28	126.5
2	2	1	4.00	134.6	2	11	0	4.51	166.0
3	2	2	3.91	121.3	3	6	0	4.54	145.0
4	9	0	4.66	167.8	4	28	1	4.42	153.8
5	29	1	4.14	143.4	5	3	0	4.19	157.4
6	17	2	3.82	131.4	6	18	0	4.28	156.4
7	29	1	3.98	113.9	7	26	1	3.78	125.3
Mean (weighted)		1.10	4.04	132.1	Mean (weighted)		0.61	4.23	146.0

[a]Percentage population of the indicated conformer in solution. Conformers having populations ≤1% have been discarded.

[b]IMHBs = intramolecular hydrogen bonds.

[c]R_{gyr} (Å) = radius of gyration.

[d]SA 3D PSA = solvent accessible 3D polar surface area.

Table S15. Summary of calculated 3D dependent molecular descriptors of the sampled conformations of compounds **1** and **2**.

	Compound **1**			Compound **2**		
Conformation No.	SA 3D PSA [b] ($Å^2$)	IMHB[c]	R_{gyr} [d] (Å)	SA 3D PSA [b] ($Å^2$)	IMHB[c]	R_{gyr} [d] (Å)
1 (MEC)[a]	113.1	2	3.7	144.2	1	3.9
2	135.6	0	3.6	114.0	2	3.8
3	116.8	1	3.9	116.6	1	4.0
4	126.0	1	4.1	152.1	0	4.1
5	145.4	1	4.1	130.7	1	4.2
6	154.9	0	4.0	154.2	0	4.4
7	140.5	1	4.0	163.3	0	4.4
8	128.6	0	4.2	138.3	1	4.4
9				152.4	0	4.3
10				146.9	1	4.1
11				136.8	1	4.4
12				144.2	1	4.3
13				137.8	1	4.4
14				137.9	1	4.2
15				165.4	0	4.5
16				151.4	1	4.3
17				160.6	0	4.3
18				134.5	2	4.2
19				155.0	1	4.4
20				160.7	0	4.6
21				156.1	0	4.5
22				139.2	1	4.1
23				142.4	0	4.2
24				125.4	2	3.8
25				114.5	1	3.7
26				143.6	0	3.8
27				163.1	0	4.5
28				139.2	1	4.3

[a]Minimum Energy Conformer

[b]SA 3D PSA = solvent accessible 3D polar surface area.

[c]IMHBs = intramolecular hydrogen bonds.

[d]R_{gyr} = radius of gyration.

Table S16. Summary of calculated 3D dependent molecular descriptors of the sampled conformations of compounds **5-7**.

	Compound **5**		Compound **6**		Compound **7**	
Conformation No.	SA 3D PSA [a] ($Å^2$)	R_{gyr} [b] (Å)	SA 3D PSA [a] ($Å^2$)	R_{gyr} [b] (Å)	SA 3D PSA [a] ($Å^2$)	R_{gyr} [b] (Å)
1	126.6	3.9	117.9	4.2	91.3	3.7
2	109.8	3.9	89.8	3.8	107.9	3.8
3	123.4	4.2	102.4	3.8	104.3	3.8
4	116.2	4.1	108.2	3.9	112.2	3.9
5	138.6	4.1	91.7	3.8	109.0	4.0
6	126.5	4.0	99.2	3.9	120.9	4.0
7	114.2	4.2	107.1	3.8	115.5	3.9
8	131.3	3.9	112.8	4.1	104.7	3.9
9	118.0	4.0	117.4	3.9	105.2	4.0
10	106.3	4.1	113.3	4.1	98.8	3.9
11	122.9	4.3	110.4	4.1	89.2	3.8
12	124.3	3.9	116.4	4.0	122.4	4.1
13	136.2	4.2	108.2	3.7	110.6	3.9
14	119.5	4.0	118.2	3.7	97.8	3.9
15	125.8	4.1	123.5	4.1	108.0	3.7
16	983	4.0	126.8	3.9	96.4	4.0
17	110.4	4.0	115.2	4.0	94.9	4.0
18	114.2	4.2	111.1	4.0	95.3	3.8
19	109.9	4.2	113.6	4.0	106.8	4.0
20	115.1	4.2	128.1	3.8	90.2	4.0
21	124.4	3.7	103.6	4.0	113.0	3.9
22	128.4	4.1	116.7	4.0	96.2	3.8
23	128.1	4.1	109.9	4.0	106.8	3.7
24	132.9	4.0	101.5	3.7	108.1	3.8
25	108.1	4.1	109.4	4.0	108.4	3.8
26	131.9	4.1	110.7	3.9	93.6	3.9
27			108.8	4.1	92.6	3.7
28			101.6	3.9	118.2	3.6
29			129.9	4.0	85.3	4.0
30			109.8	3.6	120.7	3.9
31			125.2	3.9	123.6	3.9
32			99.9	3.9	107.9	4.1
33			100.7	3.9	110.8	3.8
34			112.3	4.0	100.3	3.9
35			105.4	3.8		
36			101.8	3.9		
37			106.8	4.0		
38			92.9	4.0		

[a]SA 3D PSA = solvent accessible 3D polar surface area.

[b]R_{gyr} = radius of gyration.

Table S17. Structures, Broad Institute IDs, molecular descriptors and cell permeability values for compounds **5-7**.
Molecular descriptors were calculated using the MOE[10] software. Cell permeability and efflux ratio values were reported previously.[13]

Compound	Broad Inst. ID	Structure	MW	Permeability[a] (x10^{-6} cm/s)	ER[b]	cLogP[c]	HBA[d]	HBD[e]	TPSA[f] (Å^2)	NRotB[g]
**5**	BRD-K80841398	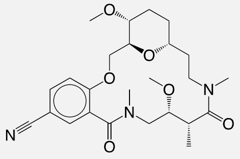	473.6	1.89	1.40	1.70	6	0	101.3	3
**6**	BRD-K29054201	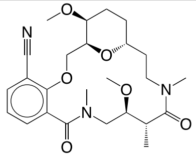	473.6	23.08	0.81	1.66	6	0	101.3	3
**7**	BRD-K33620583	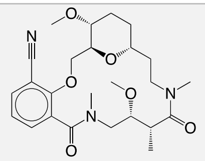	473.6	6.36	0.78	1.66	6	0	101.3	3

[a]Passive permeability across a Caco-2 cell monolayer (P_{app} AB+inhibitor cocktail); [b]Efflux ratio; [c]Calculated lipophilicity; [d]Hydrogen bond acceptors; [e]Hydrogen bond donors; [f]Topological polar surface area; [g]Number of rotatable bonds.

Principal Moments of Inertia plots

Principal moments of inertia (PMI) plots were generated for the conformations of **1**, **2** and **5-7** as described earlier.[14] The 3D-descriptors normalized principal moments of inertia ratio 1 (NPR1) and normalized principal moments of inertia ratio 2 (NPR2) were calculated using the MOE software.[10]

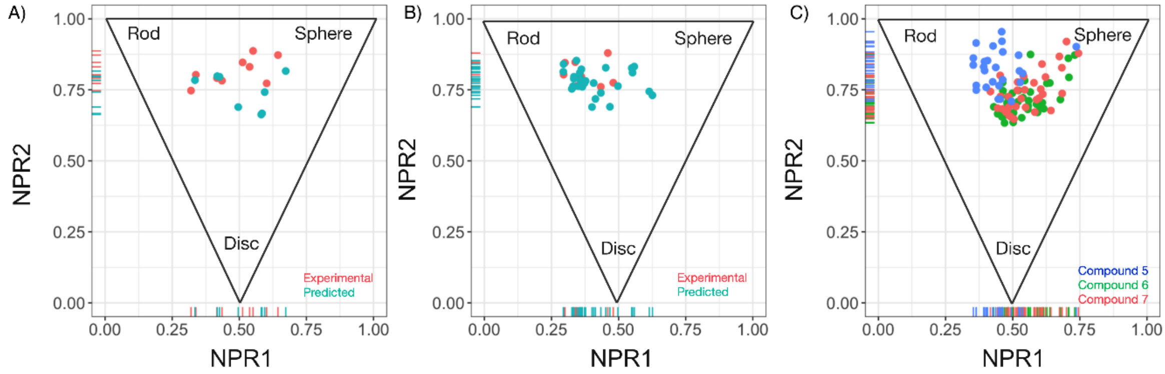

Figure S4. PMI plots for the experimentally determined and sampled conformations of compounds **1** (A) and **2** (B), as well as for the sampled conformations of compounds **5-7** (C).

Cell permeability measurement

The efflux-inhibited permeability of compounds **1-4** across Caco-2 cell monolayers was determined in the presence of a cocktail of inhibitors of efflux transporters (50 μM quinidine, 30 μM benzbromarone and 20 μM sulfasalazine) by the DMPK department at Pharmaron as reported previously.[15]

Log$D_{7.4}$ determination

Log$D_{7.4}$ was determined as reported previously.[16]

Note on synthesis, characterization and purity.

Synthesis and characterization of compounds **1-4** is reported in our previous publication.[17] The purity of compounds **1**−**4** (Figures S5-S8) was determined using a Waters LCT Premiere mass spectrometer coupled to a Waters Acquity UPLC. The Waters Acquity UPLC was equipped with either a BEH C18 column (1.7 μm, 2.1 mm × 50 mm, at 45 °C using a gradient from 5 to 90% acetonitrile modified with 40 mM ammonia and 5 mM H_2CO_3, pH 10 within 2.5 or 3 min, detection at 210 nm) or a CSHC18 column (1.7 μm, 2.1 mm × 50 mm at 45 °C using a gradient from 5 to 90% acetonitrile modified with 10 mM formic acid and 1 mM ammonium formate, pH 3, within 2.5 or 3 min, detection at 230 nm).

Synthesis and characterization of compounds **5-7** has been reported previously.[13,18]

Sample Report:

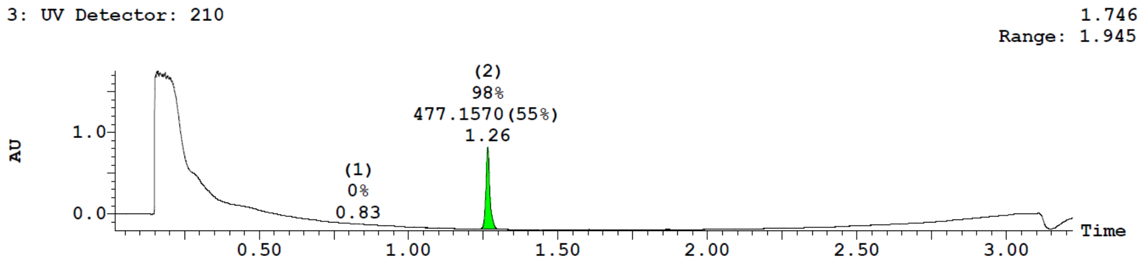

Peak Number	Compound	Time	AreaAbs	Area %Total	Height	Mass Found
1		0.83	5e+001	0.31	4e+003	
2	Found	1.26	2e+004	97.92	1e+006	477.16
3	Found	1.43	2e+001	0.11	2e+003	477.16
4		1.51	5e+001	0.30	4e+003	
5		1.60	9e+001	0.59	7e+003	
6		1.86	1e+002	0.69	9e+003	
7		2.28	2e+001	0.10	2e+003	

Figure S5. Purity analysis of compound **1**

Sample Report:

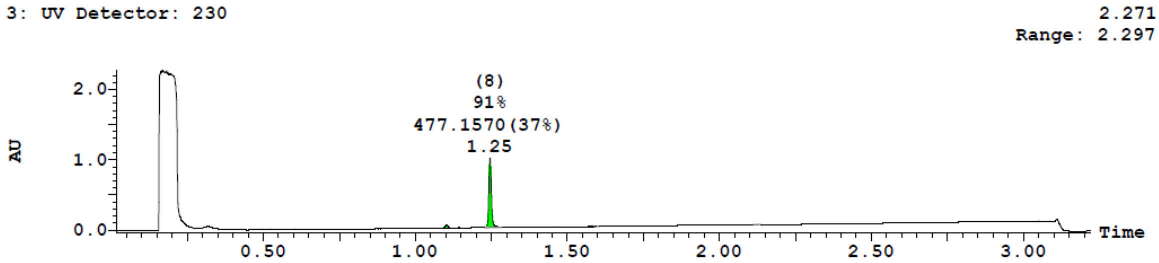

Peak Number	Compound	Time	AreaAbs	Area %Total	Height	Mass Found
1		0.68	5e+001	0.50	3e+003	
2		0.90	2e+001	0.14	1e+003	
3		0.97	3e+001	0.24	2e+003	
4	Found	1.10	5e+002	4.40	5e+004	477.16
5	Found	1.12	1e+001	0.09	1e+003	477.16
6	Found	1.14	2e+001	0.22	2e+003	477.16
7	Found	1.21	8e+000	0.07	8e+002	477.16
8	Found	1.25	1e+004	90.81	1e+006	477.16
9	Found	1.35	2e+001	0.15	1e+003	477.16
11		1.58	1e+002	0.95	9e+003	
12		1.59	5e+001	0.44	4e+003	
13		1.62	1e+002	0.91	8e+003	
14		1.65	7e+001	0.64	5e+003	
15		1.71	1e+001	0.11	1e+003	
17		2.12	3e+001	0.31	3e+003	

Figure S6. Purity analysis of compound **2**.

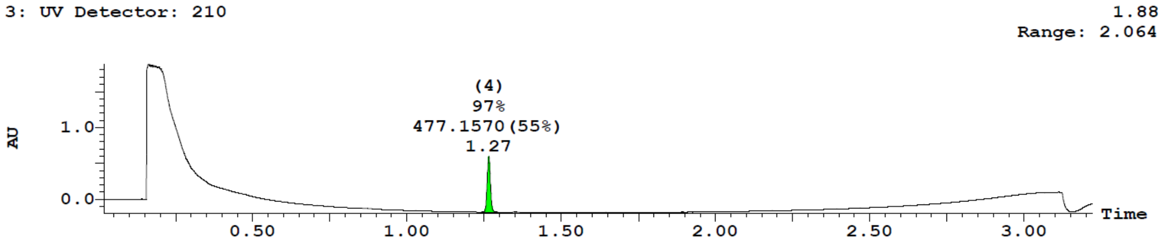

Peak Number	Compound	Time	AreaAbs	Area %Total	Height	Mass Found
1		0.87	9e+001	0.92	8e+003	
2	Found	1.21	1e+001	0.12	1e+003	477.16
3	Found	1.24	9e+000	0.09	2e+003	477.16
4	Found	1.26	9e+003	96.81	8e+005	477.16
5	Found	1.35	9e+001	0.89	9e+003	477.16
6		1.42	5e+000	0.06	9e+002	
7		1.48	2e+001	0.21	2e+003	
8		1.51	8e+000	0.08	1e+003	
9		1.56	1e+001	0.10	1e+003	
10		1.61	1e+001	0.11	1e+003	
11		1.64	5e+001	0.47	3e+003	
12		1.89	1e+001	0.15	2e+003	

Figure S7. Purity analysis of compound **3**.

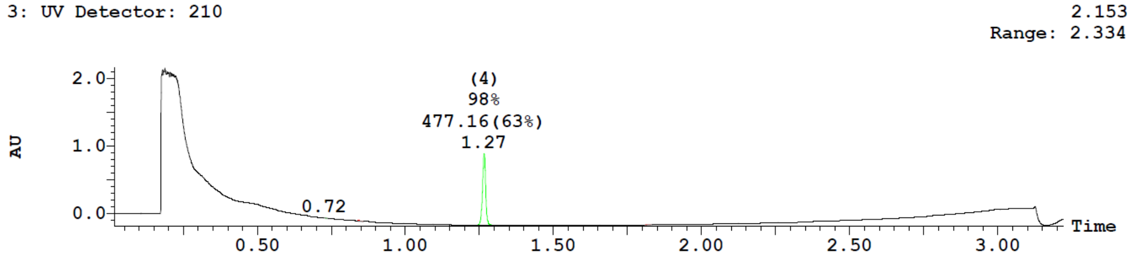

Peak Number	Compound	Time	AreaAbs	Area %Total	Height	Mass Found
1	Found	0.72	2e+001	0.11	1e+003	477.16
2		0.84	7e+001	0.49	5e+003	
3		1.03	2e+001	0.13	3e+003	
4	Found	1.27	1e+004	97.91	1e+006	477.16
5	Found	1.44	1e+001	0.11	2e+003	477.16
6	Found	1.52	5e+001	0.34	4e+003	477.16
7	Found	1.62	6e+001	0.45	5e+003	477.16
8		1.82	6e+001	0.45	5e+003	

Figure S8. Purity analysis of compound **4**.

NMR spectra

^{1}H NMR spectrum, compound **1**, $CDCl_3$

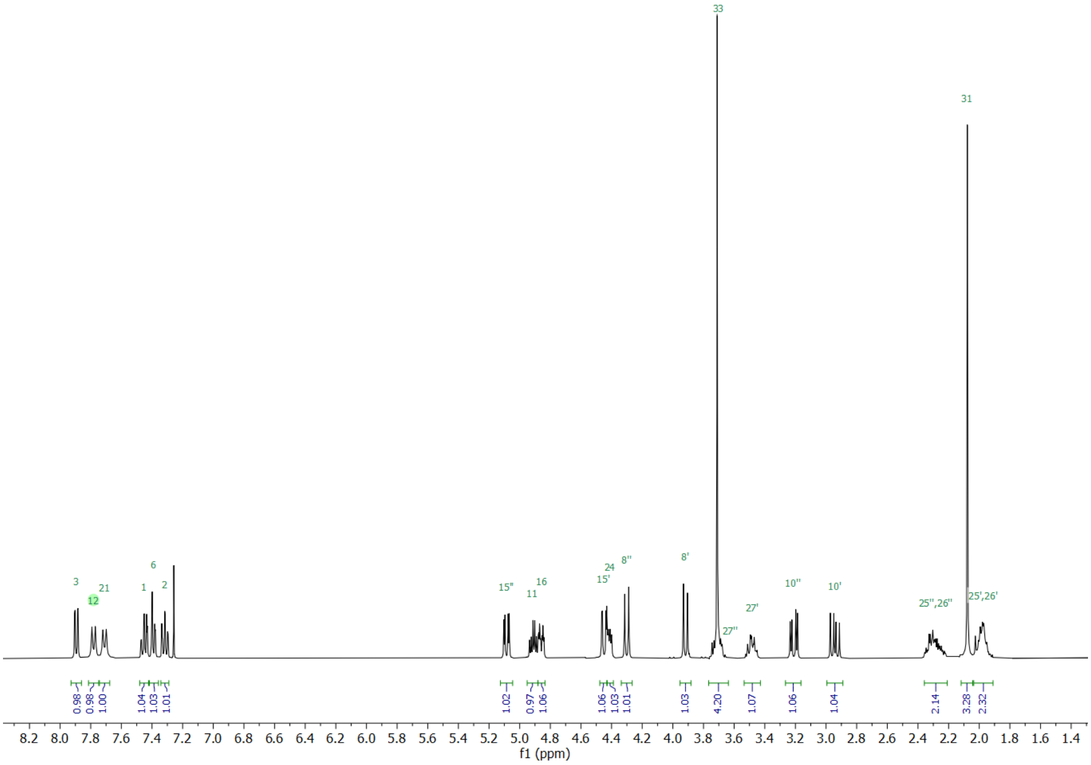

TOCSY spectrum, compound **1**, $CDCl_3$

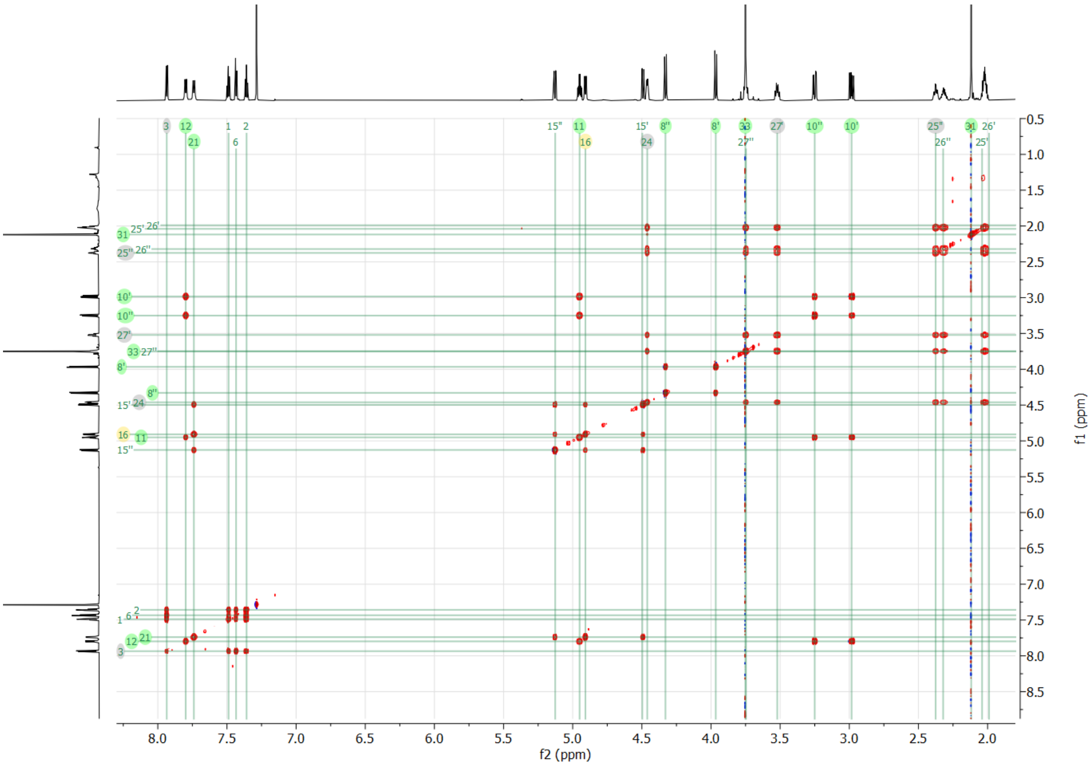

NOESY spectrum, compound **1**, $CDCl_3$

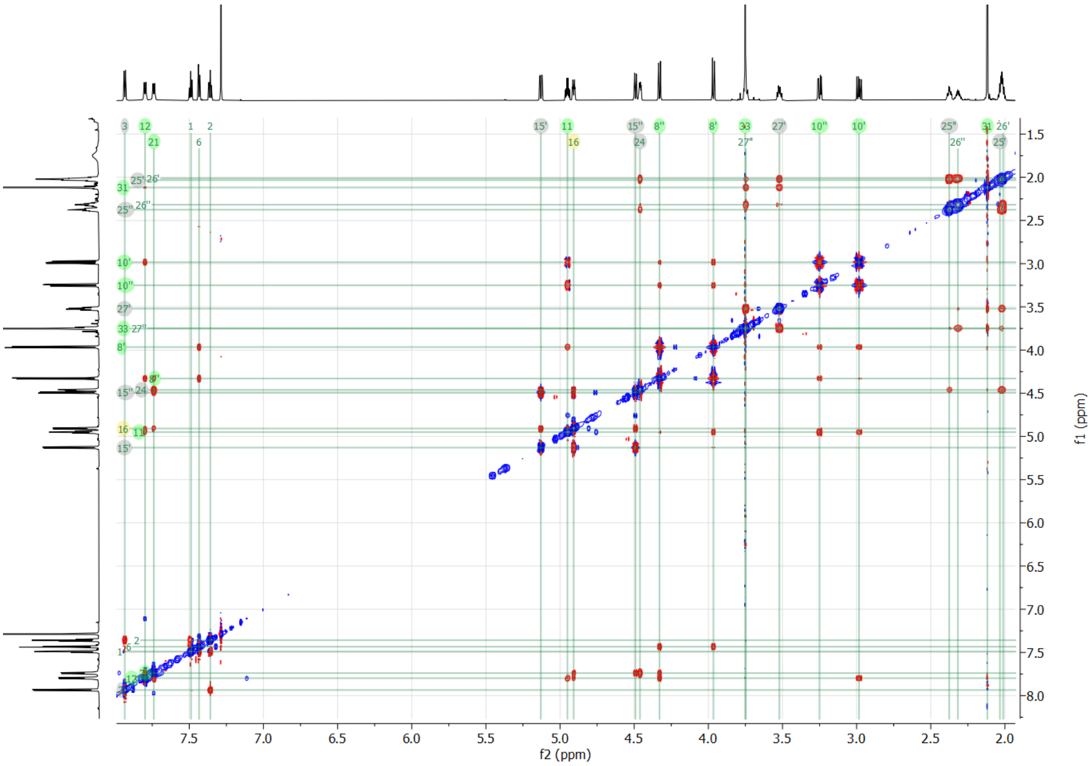

^{1}H NMR spectrum, compound **2**, $CDCl_3$

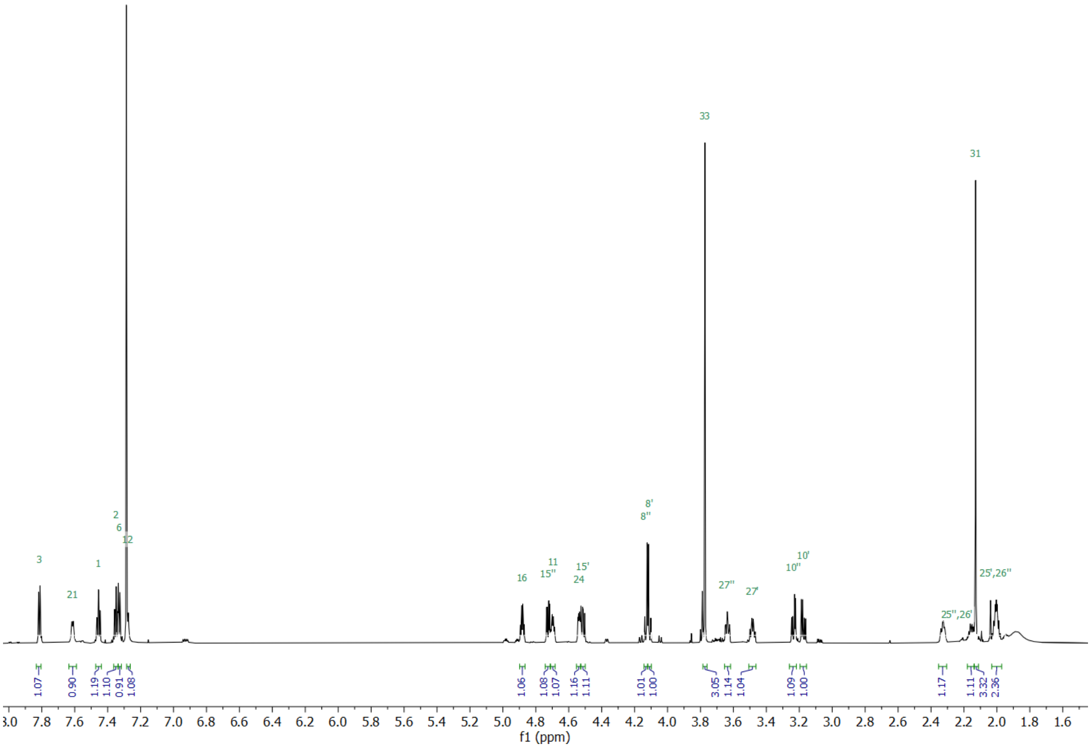

TOCSY spectrum, compound **2**, $CDCl_3$

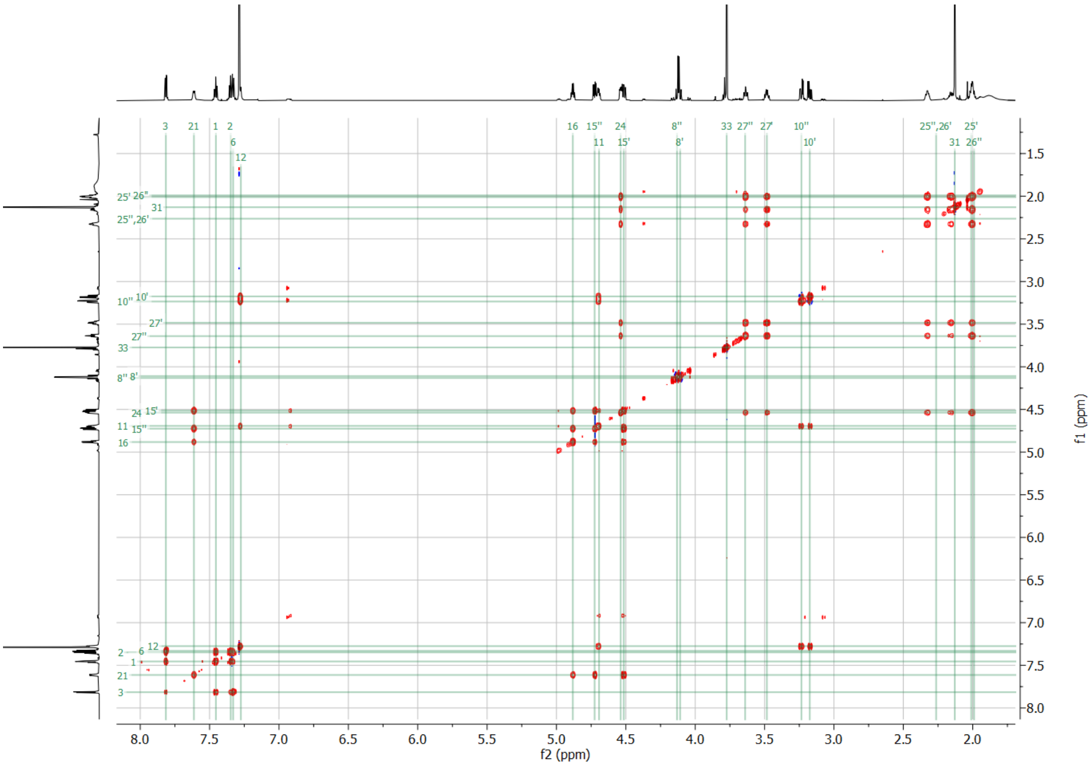

NOESY spectrum, compound **2**, $CDCl_3$

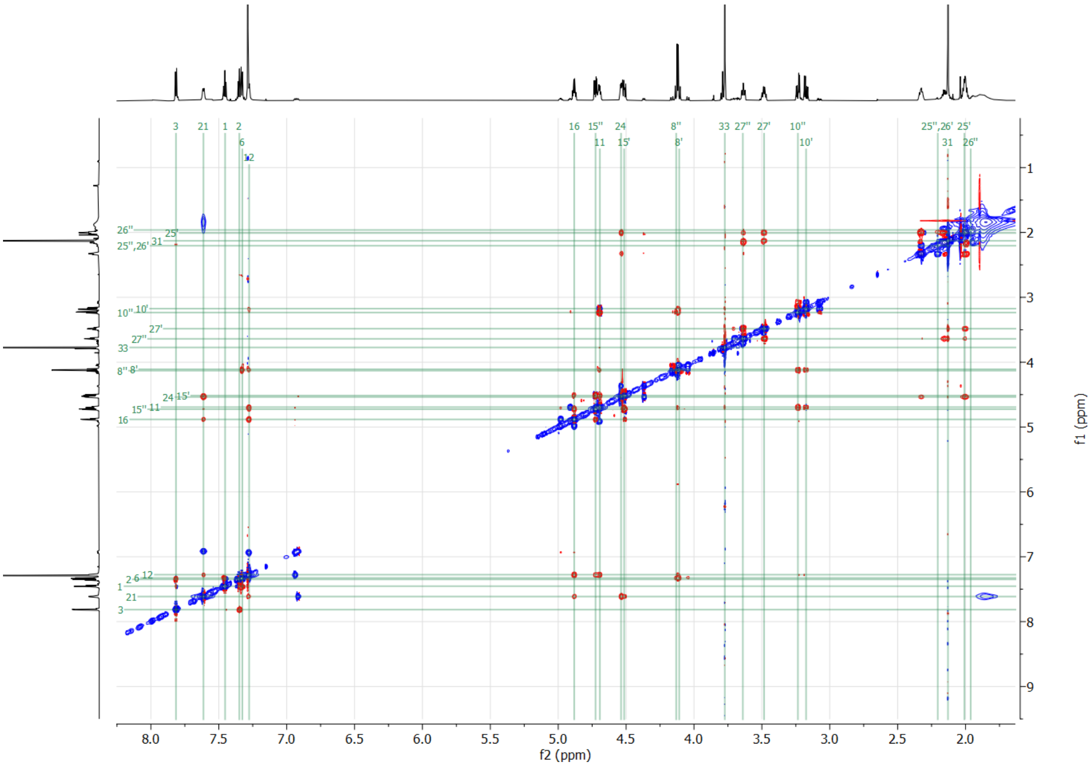

References

1. Cicero, D. O.; Barbato, G.; Bazzo, R., Nmr Analysis of Molecular Flexibility in Solution - a New Method for the Study of Complex Distributions of Rapidly Exchanging Conformations - Application to a 13-Residue Peptide with an 8-Residue Loop. *J. Am. Chem. Soc.* **1995,** *117* (3), 1027-1033.
2. Nevins, N.; Cicero, D.; Snyder, J. P., A test of the single-conformation hypothesis in the analysis of NMR data for small polar molecules: A force field comparison. *J. Org. Chem.* **1999,** *64* (11), 3979-3986.
3. Kessler, H.; Griesinger, C.; Lautz, J.; Muller, A.; Vangunsteren, W. F.; Berendsen, H. J. C., Conformational Dynamics Detected by Nuclear Magnetic-Resonance Noe Values and J-Coupling Constants. *J. Am. Chem. Soc.* **1988,** *110* (11), 3393-3396.
4. Kraszni, M.; Szakacs, Z.; Noszal, B., Determination of rotamer populations and related parameters from NMR coupling constants: a critical review. *Anal. Bioanal. Chem.* **2004,** *378* (6), 1449-63.
5. Schrödinger Release 2019-1: Maestro; Schrödinger, LLC: New York, NY, 2019.
6. Bochevarov, A. D.; Harder, E.; Hughes, T. F.; Greenwood, J. R.; Braden, D. A.; Philipp, D. M.; Rinaldo, D.; Halls, M. D.; Zhang, J.; Friesner, R. A., Jaguar: A high-performance quantum chemistry software program with strengths in life and materials sciences. *Int. J. Quantum Chem.* **2013,** *113* (18), 2110-2142.
7. Hawkins, P.C.D.; Skillman, A.G.; Warren, G.L.; Ellingson, B.A.; Stahl, M.T. Conformer Generation with OMEGA: Algorithm and Validation Using High Quality Structures from the Protein Databank and the Cambridge Structural Database *J. Chem. Inf. Model*. **2010**, 50, 572-584.
8. Grant, J. A.; Pickup, B. T.; Sykes, M. J.; Kitchen, C. A.; Nicholls, A., A simple formula for dielectric polarisation energies: The Sheffield Solvation Model. *Chem. Phys. Lett.* **2007,** *441* (1-3), 163-166.
9. Halgren, T. A., Merck molecular force field .1. Basis, form, scope, parameterization, and performance of MMFF94. *J. Compt. Chem.* **1996,** *17* (5-6), 490-519.
10. Molecular Operating Environment (2015.10), Chemical Computing Group ULC, 1010 Sherbooke St. West, Suite #910, Montreal, QC, Canada, H3A 2R7.

11. Danelius, E.; Poongavanam, V.; Peintner, S.; Wieske, L. H. E.; Erdelyi, M.; Kihlberg, J., Solution Conformations Explain the Chameleonic Behaviour of Macrocyclic Drugs. *Chem. Eur. J.* **2020,** *26* (23), 5231-5244.
12. Rossi Sebastiano, M.; Doak, B. C.; Backlund, M.; Poongavanam, V.; Over, B.; Ermondi, G.; Caron, G.; Matsson, P.; Kihlberg, J., Impact of Dynamically Exposed Polarity on Permeability and Solubility of Chameleonic Drugs Beyond the Rule of 5. *J. Med. Chem.* **2018,** *61* (9), 4189-4202.
13. Over, B.; Matsson, P.; Tyrchan, C.; Artursson, P.; Doak, B. C.; Foley, M. A.; Hilgendorf, C.; Johnston, S. E.; Lee, M. D. t.; Lewis, R. J.; McCarren, P.; Muncipinto, G.; Norinder, U.; Perry, M. W.; Duvall, J. R.; Kihlberg, J., Structural and conformational determinants of macrocycle cell permeability. *Nat. Chem. Biol.* **2016,** *12*, 1065-107
14. Sauer, W. H. B.; Schwarz, M. K.; Molecular Shape Diversity of Combinatorial Libraries: A Prerequisite for Broad Bioactivity. *J. Chem. Inf. Comput. Sci*. **2003**, *43,* (3), 987–1003
15. Fredlund, L.; Winiwarter, S.; Hilgendorf, C., In Vitro Intrinsic Permeability: A Transporter-Independent Measure of Caco-2 Cell Permeability in Drug Design and Development. *Mol. Pharm.* **2017,** *14* (5), 1601-1609.
16. Wernevik, J.; Bergstrom, F.; Noven, A.; Hulthe, J.; Fredlund, L.; Addison, D.; Holmgren, J.; Stromstedt, P. E.; Rehnstrom, E.; Lundbock, T., A Fully Integrated Assay Panel for Early Drug Metabolism and Pharmacokinetics Profiling. *Assay Drug Dev. Technol.* **2020,** *18* (4), 157-179.
17. Begnini, F.; Poongavanam, V.; Over, B.; Castaldo, M.; Geschwindner, S.; Johansson, P.; Tyagi, M.; Tyrchan, C.; Wissler, L.; Sjo, P.; Schiesser, S.; Kihlberg, J., Mining natural products for macrocycles to drug difficult targets. *J. Med. Chem.* **2021,** *64*, 1054-1072.
18. Comer, E.; Liu, H.; Joliton, A.; Clabaut, A.; Johnson, C.; Akella, L.; B., Marcaurelle, L. A., Fragment-based domain shuffling approach for the synthesis of pyran-based macrocycles. *Proc. Natl. Acad. Sci. U.S.A.* **2011**, *108* (17) 6751-6756.
